# Current Understandings of Molecular Biology of *Echinococcus multilocularis*, a Pathogen for Alveolar Echinococcosis in Humans- a Narrative Review Article

**Published:** 2015

**Authors:** Xiaoqiang WANG, Juntao DING, Xiaola GUO, Yadong ZHENG

**Affiliations:** 1 *College of Chemistry and Bioengineering, Lanzhou Jiaotong University, Lanzhou, P.R. China*; 2 *College of Life Science and Technology, Xinjiang University, Urumqi, P.R. China *; 3 *State Key Laboratory of Veterinary Etiological Biology, Key Laboratory of Veterinary Parasitology of Gansu Province, Lanzhou Veterinary Research Institute, CAAS, Lanzhou, Gansu, China*

**Keywords:** *Echinococcus multilocularis*, *Signalling pathway*, *Alternative splicing*

## Abstract

***Background:*** *Echinococcus multilocularis* is a tiny tapeworm, responsible for 0.3~0.5 million alveolar echinococcosis in humans.

***Methods:*** We searched relevant papers published between 1981 and 2013 based on the database sources such as PubMed and Google scholar, and collected and integrated the data for analysis.

***Results:*** The parasite is able to use host-originated molecules to modulate its development and has complex signalling pathways than expected previously. *E. multilocularis *utilizes many types of alternative splicing approaches to generate transcript isoforms. Recently, the genome of *E. multilocularis* has been deciphered.

***Conclusion:*** These data will give us a profound understanding of biology of *E. multilocularis*, which will promote the use as a model to study helminths.

## Introduction

*Emultilocularis*, regarded as an independent species in the 1950s (1), is a tiny tapeworm of fox with four or five segments, around 1.2~4.5mm, and completes an entire round of a life cycle via transmission between two different hosts. Alveolar echinococcosis (AE) by *E. multilocularis* is considered as one of neglected diseases*. *It is estimated that there are nearly two billion human infections of helminths in the world and *Echinococcus* contributes to the infections, around 2~3 million cases with 0.3~0.5 million AE (2). With the completion of the genome and establishment of in vitro cultivation, *E. multilocularis* has been used as a potential model for studies of helminths. 

## Methods

This search aimed to identify all valuable and relevant information considering a profound understanding of biology of *E. multilocularis*. The search covered the papers published between 1981 and 2013. Electronic searching was performed based on the international databases such as PubMed, Science Direct and Google scholar. The following key words: "*Echinococcus multilocularis* " and "Echinococcosis" were used as queries.

## Results

***Signalling pathways of E. multilocularis***

The signalling systems in animals are primarily influenced by two factors genetic heritage and living environment. Investigations of the signalling pathways in a given organism therefore allow us to understand profoundly the plasticity and evolution as well as often to develop efficient therapies. It is roughly estimated that the actions of more than 25% of commercialized medicines or drugs in the world are dependent on the pathway of G protein-coupled receptors (3). In the parasitic process, platyhelminths have evolved to communicate efficiently with hosts to harmonize their conflicts, balance immune responses and minimize the damages. Both hosts and parasites benefit from the dynamic balance that grantees parasite-induced damages to hosts at a reasonable level and in turn, to some extent, provides parasites nutrients. The activation of mitogen-activated protein kinase (MAPK) pathway in hepatic cells, which was observed especially near liver lesions in AE patients (4), reflects effects of *E. multilocularis* itself on host signalling systems. Similarly, *E. multilocularis* may take advantage of host-derived ligands, such as hormones, to facilitate infection and development (5). Parasite-parasite communications are also required to control their population size or individual size in terms of competition potentially via a conspecific parasite-parasite signalling, which is supported by a natural phenomenon of that the change of physical sizes of parasites will occur upon increased intensity at the parasitic sites. For instance, *Eurytrema *spp. is usually 10~13mm × 6~7mm in a size but this trematode becomes smaller, around 5mm × 2mm, when the population increases dramatically in the pancreatic ducts of cattle (6). 

In the evolutionary tree of animals, platyhelminths are positioned as an early emerging lineage in metazoans but they seem to lack ancestral signalling molecules and do possess the signal families (7), of which many are commonly found in mammals, demonstrating the feasible divergence of signal families before the emergence of platyhelminths. Furthermore, some signal components from the flatworms can interact with the downstream signal molecules derived from mammals, suggesting the structural and functional conservations in signal proteins from platyhelminths to mammals (5, 7, 8). As mentioned previously, the specific structures and the close contact with external environment render the tegument of parasitic platyhelminths to play important roles in signal transductions. These findings are consistent with the idea of hormonal host-parasite cross communication, which proposes the developmental functions of signal molecules of host origin by means of binding receptor tyrosine kinase (RTK) and receptor serine/threonine kinase (RSTK) on the surface of parasites, and the modulation of parasitic microenvironment induced by parasite cytokines that function after binding to host receptors (8). However, the majority of the surface molecules strongly associated with signal functions and the pathways remain to be identified. The signal networks in Echinococcus species, even in cestodes, have been largely unexplored until the nucleus genome, expressed sequence tag (EST), transcriptome and proteome projects have been launched and some of them have already finished or nearly finished. So far, the epidermal growth factor and transforming growth factor pathways in *E. multilocularis*, encompassing seventeen related genes, have been experimentally characterized (8). 

The TGF-β family has two subfamilies type I and type II, participating in a wide range of physiological regulations. The binding of ligands to TGF-β type II on the cell surface recruits TGF-β type I RSTK and then activates type I receptor kinase after the phosphorylation occurs under the actions of the ligand-TGF-β type II complex. The activation gives rise to phosphorylation of receptor-regulated Smad proteins (R-Smad) and thus the transduction of signals from outside to inside cells are fulfilled. The further transduction is executed by other Smad proteins and other proteins (7). Co-regulatory Smad (Co-Smad), 14-3-3ε, SmRK1 interacting protein (SIP) and eukaryotic initiation factor subunit 2 alpha (eIF2α), for example, appear to be involved in the TGF-β TGF pathway in *Shistosoma mansoni*, which also possesses TGF-β type I and TGF-β type II (9-12). In total, three type 1 receptors, one type II receptor and five Smad proteins have been found present in *E. multilocularis* (8). Of five Smads, four EmSmads A, B, C and E are grouped into two R-Smad subclasses TGF-β/activin (AR-Smad) and TGF-β/BMP (BR-Smad) and one EmSmad D into Co-Smad. In particular, unlike BR-Smad EmSmad B, AR-Smad proteins EmSmad A and C have no conserved domain at the N terminus that is related to DNA binding and they seem to have different activation properties. EmSmad D is capable of formation of the complex with three R-Smads that is transported into the nucleus to regulate transcription of target genes. Similar to TGF-β type I receptor of *S. mansoni* (13), *E. multilocularis* transforming growth factor EmTR1 can function together with human-derived BR-Smad to modify EmSmad B at the conserved motif via phosphorylation (14). However, in vitro studies have shown that there are no significant effects on development or phenotypic change of both flatworms when exposed to host BMP2 or TGF-β (8, 15). The mechanisms involved are still unclear and have to be clarified. One possible explanation is the existence of a feedback network that tolerates high concentration of host cytokines. It is worth to determine the real events of the TGF pathway in the period of infection. In* E. multilocularis*, the TGF pathway may link to Erk-like mitogen-activated protein kinase (MAPK) pathway via EmSmad D and to signal transduction of lipophilic hormones via the interaction of EmSmad C with a transcriptional co-regulator EmSKIP (8). 

In the EGF receptor cascade, the cytoplasmic region of phosphorylated RTK binds to such SH2 domain-containing proteins as Grb and SOS, which interact with Ras signalling pathway that activates transcription factors by a serial of cascade reactions of MAPK and corresponding kinases (7). In contrast to the insulin receptors in other animals, an insertion of 516bp is observed in the conserved tyrosine kinase domains in RTKs of tapeworms and flukes (8), which is most likely to be associated with regulation of signalling processes. The homologue to each of the EGF signal molecule families is to be encoded in *E. multilocularis*, of which the expression is in a life-long pattern. In addition, two other molecules that are potentially involved in the EGF/Ra-s/MAPK cascade, Em14-3-3 and EmPDZ-1, were also identified. Recently, Em14-3-3 and EmPDZ-1 have been found to interact with EmMKK2 that constitutes the MAPK cascade (16) and with an ezrin-radixin-moesin family component Elp (17), respectively. Several lines of in vitro evidence suggest that insulin-induced signalling is critical in early development of *E. multilocularis* larvae, and that the host-derived EGF can influence the growth and development of this parasite through the EGF/Ras/MAPK pathway (8). The ideas are compatible with the speculation that* S. mansoni* competes the EGF-like molecule of intermediate hosts which is expressed in the alumen gland in snail, *Lymnaea stagnalis*, resulting in parasitic castration (7). These findings are of great interest in the rapeutical aspect and attempts that control vesicle growth and inhibit the differentiation of neoblasts by intervention of the EGF/Ras/MAPK pathway have already been done using some specific inhibitors (8). Concerning the importance of this pathway in better understanding of the development of the parasite during infection, in vivo investigations are needed to verify the above presumptions. 

***Genomic structures of E. multilocularis***

With a few exceptions from Eucestoda and Turbellaria families, the majority of platyhelminths have no sex chromosomes and they tend to have a small number of chromosomes generally with haploid chromosomes of less than ten (7). The karyotypes of cestodes appear to be various, ranging from six to twenty. For *E. granulosus* and *E. multilocularis*, the diploid chromosome set is 2n = 18 and there are no chromosome morphological differences between these two species (18, 19). It is well known that the changes of chromosome in number and size occur during in vitro cultivation largely due to endoreduplication. After 40 passages, the cells from *E. multilocularis* larvae encompassed 14~104 chromosomes that are classified into three types telocentric, subtelocentric and metacentric but 91~100 chromosomes are dominant (20). In addition to this, the chromosome morphology tends to be related with chromosome quantity and the genome rearrangement is supposed to occur during cultivation, evidenced by morphological changes of chromosomes that are commonly discernible in cells with more than 59 chromosomes or more. Interestingly, these cells with abnormal karyotypes still exhibited infective ability to grow up into cyst masses when inoculated into cotton rats (20), suggesting the retainability of differential capacity in the cells. However, the fate of these extra chromosomes is unclear post inoculation. The dramatic alteration in a chromosome number has been also recently described in in vitro cultured cells of *E. granulosus *(21). 

The completion of the draft sequence of human genome has fuelled studies on genomes and related fields of other eukaryotic organisms. In particular, the advent of next-generation sequencing technologies, such as 454, Illumia and SOLiD that are theoretically different from Sanger sequencing approach, renders the possibilities for sequencing the entire genome in an affordable way, thus leading to the likelihood of real personalized therapy or medicine that mainly relies on individual heritage data. In flatworms, the variation of nuclear genome sizes is striking, ranging from 50Mb for Turbellaria *Stenostomum* spp. to 18,390Mb for Turbellaria* Otomesostoma auditivum *(7). However, the huge genomic divergence in size is carefully appreciated due to the presence of polypoidy that are pervasively found in *Turbellaria* species. The genome size of *E. multilocularis* is ~115 Mb, encoding 10,345 genes (22). The GC content of *E. multilocularis* genomic DNA is approximately 44% and the bias of codon usage is observed in the coding regions of highly expressed genes in *E. granulosus *(23, 24), which has also been reported in *Shistosoma *spp. (7). 

The mitochondrial genome of *E. multilocularis* is 13,738bp long with a high AC content up to 69%, comprising 2 noncoding regions and 36 genes and lacking a gene coding ATPase subunit 8 that does not exist in nematode mitochondrial DNA (25). With few or no introns or spacers, all the genes are tightly organised on the same strand, which is often conserved in the phylum Platyhelminth (7, 26). Intriguingly, two noncoding regions contain inverted repeats that are able to form hairpin structures and may function in the replication and transcription (25). 

Numerous families of repetitive sequences, contributors to a genome size, are present in parasitic flatworms investigated including Echinococcus species and they seem to be valuable in diagnosis and species identification because the repetitive sequences/elements are species- or genus-specific with regard to expression (7). *S. mansoni*, for example, possess 40% repetitive sequences that are fairly close to 40.1% in *S. japonicum*, containing 72 families of long-terminus repeat (LTR) and non-LTR transposons that account for 15% and 5% of the genome (364Mb), respectively (27, 28). There are several types of repetitive sequences present in *E. granulosus*, of which the 186bp-long TREg element is genus-specific at strain-dependent copies from 120 to 23,000, while the EgG1 Hae III is species-specific, dispersed in the genome with approximately 7,000 copies (29-31). However, the functions of these repetitive elements remain elusive. 

***Gene expression of E. multilocularis***

Spliced-leader *trans*-splicing (SL *trans*-splicing) is an ancient eukaryotic feature, which was first described in *Trypanosoma brucei* and subsequently found in other organisms including Euglenozoa, Turbellaria, Trematoda, Cestoda, Nemathelminthes, Chordates and Cnidaria (32). The proportion of RNA that is transcriptionally modified via SL *trans*-splicing is various. It is estimated that SL-containing mRNA accounts for 10%~20% in *S. mansoni* (33), while the percentage reaches up to 70 in *Caenorhabditis elegans *(34). It is well known that SLs are absolutely conserved in nematodes but not in trematodes. Homological analysis of SLs in platyhelminths supports the idea of the common ancestral origin (7). 

In the phylum Platyhelminth, SL *trans*-splicing are present in all Digenea species investigated so far but absent in Monogenea (7). In Cestoda and Turbellaria, this RNA editing approach seems to be complicated and numerous studies have shown that some parasites possess this type of splicing but the others not, presumably suggesting the independent loss or gain of the SL trans-splicing in these lineages (32). The gain of SL RNAs might happen from an Sm-binding U snRNA or from a part of a gene that has the promoter, the first exon and part of the first intron, and the dysfunction of the SL* trans*-splicing can result from the depletion of the SL RNA or a key constituent for a SL small ribonucleoprotein particle. There is to date no direct evidence to crystallize the relationship between the gain or loss of SL *trans*-splicing and evolution in cestodes and planarias. The finding that SL *trans*-splicing is present in chordates supports the idea that vertebrates might have lost this mRNA editing mechanism during evolution (35), indirectly evidenced by the fact that *C. elegans* SL RNA can be utilized to *trans*-splice in mammalian cells (36), and the SL *trans*-splicing lack may have been driven by genome expansion (32). 

The presence of SL *trans*-splicing in *E. multilocularis* was firstly identified by the facts that there are two elp transcripts that differ from the first small exons that are not likely to reside at the same locus (37). *E. multilocularis* SL and SL RNA are 36bp and 104bp in size, respectively, with 97% and 98% similarity of that of *E. granulosus*. The SL RNA-coding gene copies are considerably lower in *E. multilocularis* than these in trematodes and nematodes. 25% of *E. multilocularis* mRNA, approximately 4,600 transcripts of which 40% exhibit differential expression, are estimated to undergo SL *trans*-splicing but the profile of SL *trans*-spliced mRNA illustrates no correlation between *trans*-spliced mRNA and corresponding protein functions (38). In addition, the SL *trans*-splicing shows no tissue- or stage-specific features, which has also been reported in trematodes (7, 38). 

Alternative splicing (AS), inclusion of different exons of a mRNA precursor (pre-mRNA) during maturation, is an essential editing event in multicellular organisms, by which more than 90% human genes are modified (39). AS is considered present in almost the entire eukaryotic kingdom but AS events are more in higher eukaryotes than lower eukaryotes and alternatively spliced genes are more in vertebrates than invertebrates. Moreover, AS shows tissue or stage specificity and is common among regulatory protein-coding genes (40). AS comprises of four types exon skipping, intron retention, and alternative 5’ splice site (SS) and 3’ SS selection. It is worthwhile mentioning that the SL *trans*-splicing described above is a rare and special type of AS. Exon skipping that accounts for ~40% of AS events is dominant in higher organisms, while intron retention (around 30%) prevails in plants, fungi and protozoa (41), demonstrating extraordinary differences of distinct AS types in contribution to phenotypic complexity. It is noted that our growing and profound understanding of AS has been benefiting from extensive applications of cutting-edge approaches including splicing-sensitive microarray, next-generation sequencing technologies and other genome wide studies. Generally AS results in mRNA variants that encode protein isoforms with different functions and premature stop codon-containing RNA species that are subsequently degraded via a nonsense-mediated mRNA decay pathway (42). A large number of mRNA variants that are synthesized through AS, to a large extent, fills the gap between human gene number and different proteins that are predicted to be produced (43). The occurrence of alternatively-spliced mRNA with premature stop code is at a low frequency. 75% of AS can influence coding regions of genes, mostly loop or coiled parts on the surface of corresponding proteins that may alter interactions between proteins, location or/and catalytic capacity (40, 44). A mounting body of studies has shown the strong connections between aberrant AS and human diseases including cancers in which mutations in an exon, intron, or splicing donor or acceptor site of tumor suppressor genes occur and adversely affect the production of normal transcripts (42, 45). 

In cestodes, exon-skipping splicing was firstly reported in *Taenia* *solium*, a causative agent of cysticercosis (46). It is also reported that both *E. granulosus* and *E. multilocularis* can skip alternative exon (s) to generate different mRNA transcripts from a single multi-exon pre-mRNA (47, 48). *E. multilocularis* mitogen-activated protein kinase1-coding gene (emmpk1), for instance, contains 10 introns, dispersing on a 6.7kb chromosomal locus. In the splicing, introns from 1 to 8 and 10 are completely removed and another two alternative splicing acceptor sites in intron 9 are utilized, allowing intron fragments to be served as coding sequences and leading to formation of three different molecules that are constitutively expressed in metacestodes, invaginated protoscoleces and activated protoscoleces. 

Strikingly, *Echinococcus *species can combine different AS patterns, such as exon skipping and SL *trans*-splicing, to expand protein diversity from an individual gene. The transcription of thioredoxin glutathione reductase (TGR) in *E. granulosus* exemplifies this concept. There exist two variants of TGR that are equally expressed but localized in mitochondrion and cytoplasm, respectively (47). With combined use of SL *trans*-splicing and exon skipping, removal of exon I that offers a signal for mitochondrial translocation makes difference between these TGR transcripts that are supposed to be translated into the same proteins. Two potential mechanisms were postulated to be involved in the splicing but the presence of a transcription initiation site in intron 1 suggests that two TGR mRNA molecules may be derived from different pre-mRNA transcripts. This combined use of different AS that yields different transcripts was also described in *E. multilocularis* (37). There is no direct evidence to illuminate whether or not exon skipping occurs before SL *trans*-splicing in the process of mRNA editing. Moreover, it is not clear whether mRNA variants are pervasively produced in an approach of combined AS types in *Echinococcus* parasites. 

## Conclusion

Concerning the importance of signalling pathways during parasite infection, in vivo investigations are needed to verify the above presumptions. With the completion of the genome and establishment of *E. multilocularis*, it has been used as a potential model for studies of helminths.
